# The Role of Selection for Function in Aging and Chronic Diseases: A Novel Evolutionary Perspective

**DOI:** 10.1111/acel.70207

**Published:** 2025-09-05

**Authors:** Antoine M. Dujon, Klara Asselin, Jean François Lemaître, Jean‐Pascal Capp, Pascal Pujol, Beata Ujvari, James DeGregori, Aurora M. Nedelcu, Frédéric Thomas

**Affiliations:** ^1^ CREEC/CANECEV, MIVEGEC (CREES) Department University of Montpellier, CNRS, IRD Montpellier France; ^2^ School of Life and Environmental Sciences Deakin University Waurn Ponds Victoria Australia; ^3^ CNRS, UMR5558, Laboratoire de Biométrie et Biologie Évolutive Université Lyon 1 Villeurbanne France; ^4^ Toulouse Biotechnology Institute University of Toulouse, INSA, CNRS, INRAE Toulouse France; ^5^ Oncogenetic Department University Medical Centre of Montpellier Montpellier France; ^6^ Department of Biochemistry and Molecular Genetics University of Colorado Anschutz Medical Campus Aurora Colorado USA; ^7^ Department of Biology University of New Brunswick Fredericton New Brunswick Canada

## Abstract

Aging, and by extension age‐related diseases, has traditionally been understood through classical evolutionary genetic models, such as the mutation accumulation and antagonistic pleiotropy theories. However, these frameworks primarily focus on the declining efficacy of organismal‐level selection against mutations with deleterious effects in late life. Here, we propose a novel hypothesis: many chronic diseases associated with aging may emerge, at least in part, as a result of selection acting at lower organizational levels, including non‐replicative biological entities, enabled by the relaxation of selective pressures that constrained within‐organism evolutionary processes in early life. This hypothesis is built on the recently proposed concept of selection for function that extends the evolutionary process to non‐replicative entities. While Darwinian selection acting at the organismal level strongly constrains within‐organism evolution during an organism's reproductive lifespan, these constraints weaken with age. As a consequence, lower‐level non‐replicative entities, such as benign and malignant tumors, atherosclerotic plaques, and neurodegenerative aggregates, may experience a form of selection that favors those with increased stability, organization, and long‐term persistence, sometimes at the cost to host fitness. These entities do not evolve via long‐term differential reproduction, but rather certain configurations of their structure persist preferentially over others due to environmental constraints, microenvironmental selection, and internal stabilization mechanisms. Understanding aging through the lens of selection for function at the level of internal non‐replicative entities provides new insights into the evolution of chronic diseases and opens novel therapeutic avenues aimed at disrupting internal functional organization, rather than merely targeting cellular proliferation/abnormalities or disease symptoms.

## Graphical abstract

This conceptual diagram shows how aging alters selection within the body: strong early‐life Darwinian selection prevents aberrant structures, while weakened selective pressure in later life allows persistent damage‐related entities to stabilize. These may reorganize internally, becoming resistant, under a late‐life “selection for function” that favors local persistence over systemic integrity.

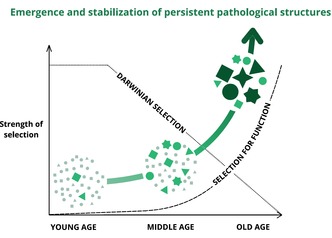

BOX 1 **The concept of selection for function.**

Selection for function is an emerging evolutionary framework that explains how certain structural or organizational configurations—biological or not**—**persist over time, regardless of their reproductive capacity (Wong et al. 2023). Unlike classical Darwinian selection, which operates through differential reproductive success of self‐replicating entities, selection for function favors configurations that enhance the persistence, robustness, and adaptability of a system as a whole.

This mechanism, recently formalized by Wong et al. (Wong et al. 2023), is based on three key principles:

1. Static persistence: Some configurations remain stable because they resist degradation or disruption.
2. Dynamic persistence: Systems can adapt to perturbations, increasing their resilience without replication.
3. Novelty generation: New configurations emerge that confer greater system‐level stability or functionality.

A system evolves through selection for function if it meets the following criteria:

- It consists of many interacting components capable of forming a wide range of configurations.
- Mechanisms exist that generate diverse configurations.
- Certain configurations are preferentially stabilized due to their contribution to the persistence of the system.

This concept applies not only to biological systems but also to abiotic systems (e.g., mineral formations), technological systems, and even ecological networks. Within organisms, non‐replicative structures—such as atherosclerotic plaques, amyloid aggregates, or benign tumors—may evolve toward increasingly organized forms via selection for function, particularly when higher‐level constraints (e.g., organismal fitness control) decline with age.

By shifting the evolutionary focus from replication to persistence, selection for function offers a unifying principle that expands the scope of evolutionary theory beyond gene‐centric models, providing a novel lens through which to understand the evolution of chronic diseases in aging organisms.

Aging, the progressive decline in physiological condition and performance, is traditionally seen as the principal risk factor for morbidity and mortality in humans (Niccoli and Partridge 2012). Advancing age is generally accompanied by a marked increase in diseases such as cancer, neurodegenerative disorders, and chronic inflammatory conditions (Siegel et al. 2024; Montégut et al. 2024; Haikal and Weissert 2024; Stephen 2015; Andonian et al. 2024), ultimately contributing to the age‐specific decline in both reproductive and survival probabilities (Partridge and Barton 1993). Canonical evolutionary genetic theories seeking to explain the origin and evolution of the aging process rely on the progressive decline in the strength of natural selection against deleterious alleles with late‐in‐life effects from sexual maturity onwards, leading to what has been termed “selection shadow” (Hamilton 1966). Depending on the overall fitness effect of these alleles (and the evolutionary forces responsible for their fixation), two distinct (though not mutually exclusive) evolutionary theories have been proposed. The mutation accumulation (MA) theory (Medawar 1952) posits that across many generations this decline in the ability of natural selection to eliminate mutations with late‐onset detrimental effects creates conditions for the passive/neutral accumulation of late‐in‐life deleterious mutations in a population, whose late‐in‐life expression at the individual level reflects in a deterioration of health. On the other hand, the antagonistic pleiotropy theory (AP) (Williams 1957; Gaillard and Lemaître 2017; Austad and Hoffman 2018) posits that mutations with detrimental effects in late‐life can actually be selected for (i.e., positive selection) if they confer fitness benefits during early life through an acceleration of the growth process or an increase in reproductive performance. Compelling evidence from both laboratory and wild studies suggests that genes with antagonistic pleiotropic effects contribute to the aging phenotype (Austad and Hoffman 2018; Nussey et al. 2013), and in some cases, such genes have been directly linked to the occurrence of late‐onset diseases (Byars and Voskarides 2020). A classic example is the APOE gene (coding for a protein involved in lipid transport), in which the ε4 allele increases the risk of cardiovascular diseases, dementia, and Alzheimer's disease (Raichlen and Alexander 2014), while providing fertility advantages for women carrying at least one copy of this allele (Jasienska et al. 2015; Trumble et al. 2023).

In addition to these population genetic models, the disposable soma theory (Kirkwood 1977) proposes that aging may primarily result from the progressive relaxation of selection on somatic maintenance after reproduction, leading to reduced investment in the upkeep of the soma. Consequently, at a mechanistic level, aging results from a progressive accumulation of unrepaired damages in the organism due to a resource‐based allocation trade‐off between growth and reproduction on the one hand, and somatic repair on the other hand (Kirkwood 1977). Similarly, the developmental theory of aging suggests that differences in the optimal level of gene expression between early and late‐life stages contribute to aging. Thus, selection will favor near‐optimal gene expression during development and early adulthood, but will not be able to adjust this expression in late‐life, when organisms enter the selection shadow (Blagosklonny 2006; De Magalhães and Church 2005; Gems 2022). These two non‐mutually exclusive theories emphasize life‐history trade‐offs as the cornerstone driver involved in the evolution of aging (Maklakov and Chapman 2019; Lemaître et al. 2024). Importantly, these theories can encompass most of the hallmarks of aging (e.g., deregulating nutrient sensing pathways and telomeres attrition; see (Lemaître et al. 2024)), a set of key molecular mechanisms driving the aging process and contributing to the prevalence of age‐related diseases (López‐Otín et al. 2013, 2023).

Regardless of the evolutionary theory invoked, the general dysfunction of the aging organism visible at various levels of biological organization (e.g., cells, tissues or organs), and responsible for the occurrence of multiple late‐onset diseases (Niccoli and Partridge 2012), is the result of the age‐specific decline in the ability of natural selection to eliminate deleterious alleles with late‐in‐life effects. In these scenarios, evolutionary processes act at the level of the individual to either “allow” such alleles to passively accumulate in the population (as they do not detrimentally affect fitness during early life or reproductive years; thus, cannot be eliminated/purged) or to facilitate their fixation because of their positive effects on early growth and reproduction. However, selection can also act at levels below that of the multicellular organism and can affect the fitness of both the lower and the higher levels. This is most obvious during the initiation and progression of cancer when cells with mutations that increase their fitness (survival and proliferation) are selected for (Ujvari et al. 2017). Importantly, while genetic mutations have long been emphasized, stable epigenetic alterations, such as DNA methylation or histone modifications, can similarly drive heritable changes in gene expression that affect cell behavior and tissue structure. Such epigenetically driven configurations, even in the absence of DNA sequence changes, may also be subject to selection pressures (Mc Auley 2023). Nevertheless, a new concept, “selection for function”, has recently been proposed (Wong et al. 2023) (see next section and Box 1) to extend the scope of natural selection to non‐replicative systems and account for their evolution, particularly in terms of increased complexity. Here, we are addressing whether aging and age‐related diseases might reflect evolutionary processes that involve lower‐level non‐replicative entities.

The current set of theories that account for the multiple evolutionary mechanisms underpinning aging often treat pathology as a passive process, one driven by the failure of maintenance mechanisms (as a result of decreased selection strength in late life), rather than acknowledging the active organizational properties of many chronic diseases, with the notable exception of cancer, which has long been considered an active within‐organism evolutionary process. However, in addition to cancer, a key feature of several age‐related diseases is that they do not merely arise from tissue degradation, but instead display structured, self‐organizing properties that contribute to their persistence. For instance, atherosclerotic plaques are not static lipid accumulations, but highly organized biological structures, where immune cells and vascular smooth muscle cells reinforce the fibrotic cap, stabilizing the plaque (Wu and Zhang 2024). In neurodegenerative diseases such as Alzheimer's, amyloid‐beta plaques and tau tangles actively disrupt neuronal clearance mechanisms, resisting degradation and persisting as stable aggregates. While many precursor structures may initially form, only those configurations that resist clearance and persist over time accumulate and contribute to disease progression. These processes resemble selection, even in the absence of replication of individual entities (i.e., plaques), and we suggest that their selection is likely favored during late‐life, within the selection shadow (Xin et al. 2018).

## 1 Selection for Function and the Evolution of Internal Entities

Selection for function (see also Box 1), recently proposed by Wong and colleagues (Wong et al. 2023), describes an evolutionary mechanism that favors the persistence, optimization, and increasing complexity of structural and functional configurations of systems, regardless of their reproductive abilities. The evolution of such systems operates through three key principles: *static persistence*, which stabilizes configurations and prevents their degradation; *dynamic persistence*, which enhances system robustness by enabling adaptation to perturbations; and *novelty generation*, which fosters the emergence of new configurations that improve system‐level resilience. These processes result in the evolution toward increased complexity of the system. Darwinian selection can be viewed as a specific case of selection for function that applies to biological systems with self‐replicative abilities; in this context, selection for function refers to selection for increased fitness, adaptability, and evolvability.

While we adopt the terminology *selection for function* as defined by Wong et al. (Wong et al. 2023), we recognize that the use of the word *function* may be potentially misleading, especially in the context of chronic diseases and aging, where this term has been employed multiple times (e.g., (Gems 2022; Maklakov and Chapman 2019)). In this framework, *function* does not imply a benefit at the level of the organism. Instead, it refers to the capacity of internal structural configurations, such as plaques or aggregates, to enhance their own persistence, internal organization, or resistance to degradation within the aging body. These “functions” may support the stability of these entities through local interactions (e.g., with immune cells, the extracellular matrix, or metabolic flows), but often at the expense of organismal fitness. Thus, while we retain this terminology for consistency with the original formulation by Wong et al. (Wong et al. 2023), we emphasize that it does not equate to adaptive function in the classical evolutionary sense.

Unlike Darwinian selection, which acts primarily on self‐replicating entities through differential reproductive success, selection for function operates more broadly, shaping populations, ecosystems, and even non‐biological systems, such as minerals (e.g., (Hazen and Wong 2024)), where stability, adaptability, and novelty generation drive evolution over time. By shifting the focus from reproduction, selection for function extends evolutionary theory beyond the realm of biological life, providing a unifying framework for understanding the emergence, maintenance and evolution of complex systems across multiple levels of organization, from molecular assemblies to large‐scale abiotic systems. Wong and colleagues (Wong et al. 2023) argue that all evolving systems are similar in that they display three notable characteristics: “(1) they form from numerous components that have the potential to adopt combinatorially vast numbers of different configurations; (2) processes exist that generate numerous different configurations; and (3) configurations are preferentially selected based on function”, that is, the ability of the configuration to increase the persistence of the system displaying it. Each system subject to selection for function evolves via the selection of advantageous configurations with respect to system‐level persistence. In this framework, any system that meets these criteria has the potential to evolve.

We emphasize here that selection for function may also operate within individual organisms, shaping the persistence and organization of internal non‐replicative biological systems. However, unlike traditional selection and evolutionary processes, this does not imply an evolutionary trajectory based on the differential reproduction of these structures, but rather a differential filtering of specific configuration states that maximize resilience in a given physiological or pathological context.

The persistence and evolution of such structures suggest that two evolutionary forces (acting at two levels) can interact to shape aging‐related pathologies. First, selection for function can act on specific configurations of internal components associated with a particular pathology. Among lower‐level non‐replicative biological structures, certain spatial and structural organizations may emerge as more stable and resilient, thereby favoring their persistence, regardless of their impact on the higher level. For example, in atherosclerosis, certain plaque structures, like fibrous caps, resist clearance (i.e., are “selected for”) and thus promote lesion persistence (i.e., a pathology). Supporting the relevance of selection processes acting on non‐replicative units, particularly in lesions such as atherosclerotic plaques and neurodegenerative aggregates, there is growing evidence that lesion persistence and progression are highly variable, an essential prerequisite for selection to operate. Note that in these cases, the variation is not heritable and is not due to replication processes; rather, these structures emerge *de novo* and self‐assemble. Many early structures are actively cleared or regress, indicating that only certain configurations are maintained over time, an essential prerequisite for selection for function to operate. In atherosclerosis, for instance, the dynamic balance between stabilizing and regressive plaques has been extensively documented, with some lesions undergoing structural remodeling and inflammation‐dependent regression (e.g., (Watson et al. 2020; Libby 2021; Tabas and Bornfeldt 2016; Itabe et al. 2011)). Similarly, in Alzheimer's disease, early‐stage amyloid‐beta or tau aggregates can be cleared by glial cells, and their persistence depends on specific microenvironmental conditions and structural conformations (e.g., (Spires‐Jones and Hyman 2014; Rahman and Lendel 2021; Wyss‐Coray and Rogers 2012)). These observations strongly suggest that structural persistence is not universal and that some configurations are selectively stabilized, a hallmark feature of selection for function.

**FIGURE 1:**
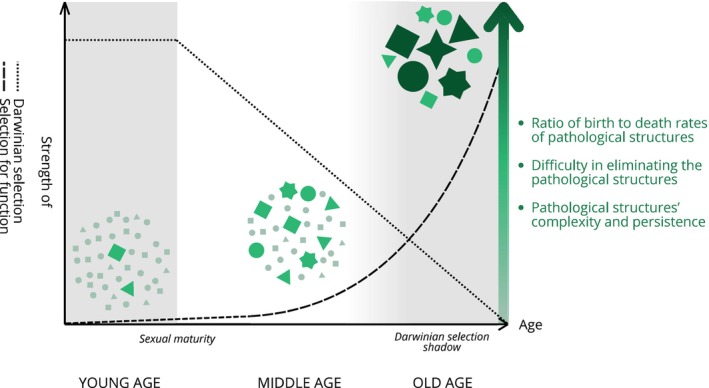
Conceptual illustration of the emergence and stabilization of persistent pathological structures via selection for function across the lifespan. While classical evolutionary theories of aging have largely focused on the decline of Darwinian selection with age (dotted line), our perspective suggests that this relaxation does not simply allow for the accumulation of genes with age‐specific deleterious effects, but rather opens the door for alternative selective forces to operate within internal biological systems (dashed line). The graphic indicates the global shapes of the curves, but their slopes and limits are arbitrarily chosen. We used those shapes for simplicity reasons, although there are other factors that temper the strength of selection. At young ages (left), most structures are transient (small size pale green forms), poorly organized, and rapidly eliminated. With aging (middle), clearance mechanisms weaken, allowing certain structures (middle size green forms) to persist. By old age (right), only a subset of entities with specific properties, such as compactness, internal organization, or resistance to degradation, remains and further consolidates (big size dark green forms), reflecting their functional persistence. These increasingly dominant forms result from internal selective‐like dynamics, rather than random accumulation.

Second, Darwinian selection acting at the organismal level, which would normally act to suppress such evolutionary processes at the lower level (analogous to suppressing cancer) when they threaten survival, progressively loses its efficacy from sexual maturity onwards, allowing selection for function to operate at the lower level with fewer constraints (Figure 1). During development and the reproductive stages, natural selection not only promotes traits that maximize growth and reproductive success, but also constrains the potential for the evolution of non‐replicative entities, resulting in functionally persistent yet potentially deleterious biological structures. This constraint limits the ability of selection for function at the lower levels to generate overly stable internal networks that could compromise the organismal health of the higher level (the organism). However, after sexual maturity, as the force of natural selection declines (Hamilton 1966), these constraints progressively weaken, allowing selection for function to re‐shape internal systems with fewer restrictions (Figure 1). This relaxation does not lead to the evolution of entirely new systems, but rather enables the preferential persistence of specific configurations within existing biological structures, promoting increased organization and resistance to degradation over time. In parallel, aging tissues experience a decline in cell turnover and plasticity due to stem cell exhaustion and epigenetic drift, including a progressive loss of epigenetic information (Yang et al. 2023; Bertucci‐Richter and Parrott 2023). These mechanistic changes reduce the regenerative capacity of tissues, creating an internal environment more favorable to the stabilization of pathological structures. In neurodegenerative diseases and atherosclerosis, for example, the depletion of neural and mesenchymal stem cells may facilitate the persistence of aggregates or plaques shaped by selection for function. This shift may underlie the progressive rise of chronic age‐related diseases and exacerbate the aging phenotype, as internal biological structures become increasingly self‐organized and resistant to regulation (Figure 1), sometimes with detrimental effects on organismal fitness. We propose that this dual mechanism explains why many chronic diseases are not merely degenerative but instead exhibit increasing structural complexity, making them more resistant to therapeutic interventions.

## 2 The Role of Selection for Function in Cancer and Other Age‐Related Chronic Diseases

It has recently been proposed that tumors exemplify how selection for function at the sub‐organismal level can drive the persistence and progression of pathological entities (Thomas et al. 2024). This hypothesis challenges the conventional Darwinian view of tumorigenesis, which attributes cancer progression solely to natural selection acting on individual cells and cell groups based on their differential reproductive success (e.g., (Cairns 1975; Nowell 1976)). While Darwinian processes acting at the cell level undoubtedly generate genetic and phenotypic diversity within tumors, they alone are insufficient to explain why some tumors progress while others remain stable or regress. Instead, tumor evolution appears to be also shaped by selection for function, which acts at the tumor level rather than exclusively at the level of individual cells (Thomas et al. 2024). However, tumors do not evolve as standalone units. Rather, they exhibit dynamic changes in their group phenotypic composition (GPC), meaning that particular cellular and structural configurations persist preferentially under certain microenvironmental conditions (Capp et al. 2021). Tumors that acquire an oncogenic GPC aligned with their microenvironmental conditions at a given stage and host age are more likely to persist and progress. This persistence does not rely on classical genetic inheritance but instead emerges from the optimization of functional configurations/organizations, where factors such as mechanical constraints, metabolic adaptations, and immune evasion contribute to the stabilization of the GPC.

It is therefore important to distinguish selection for function from classical Darwinian adaptive processes in cancer evolution. While Darwinian selection primarily acts on genetic and phenotypic variation within cell populations, selection for function operates at a higher organizational level. Yet, this process is distinct from group selection (involving group reproduction) as tumors are not reproducing units with heritable properties. Instead, selection for function favors tumor configurations that persist despite environmental fluctuations. This distinction has key therapeutic implications: it suggests that treatments should not only target cellular heterogeneity, but also aim to disrupt the emergent functional networks that sustain tumor growth. By stabilizing particular structural configurations, selection for function enables tumors to develop robust communication networks, metabolic adaptations, and cooperative interactions that contribute to treatment resistance. Recognizing selection for function as a key driver of tumor evolution thus opens new therapeutic avenues. While current cancer treatments typically aim to eradicate malignant cells, this approach often overlooks the role of functional organization in tumor persistence. Strategies focused on disrupting the tumor's functional architecture, rather than solely targeting individual cancer cells, may prove more effective in controlling tumor progression (Thomas et al. 2024). Furthermore, this perspective extends beyond malignant tumors: selection for function may also explain the persistence and increasing complexity of certain benign growths in aging individuals, such as polyps, cysts, and lipomas (Boutry et al. 2022). Like malignant tumors, these structures may undergo a process of functional reinforcement, where local selective pressures (e.g., nutrient gradients, inflammatory responses, mechanical constraints) guide their organization toward structurally stable configurations.

Interestingly, pediatric cancers may represent a counterexample to this pattern. Unlike many adult cancers that benefit from selection for function at the tumor level, allowing them to establish stable and structured configurations, pediatric malignancies typically emerge and progress rapidly. The tissues most affected in childhood cancers (blood, bone, and brain) are characterized by high developmental plasticity and rapid cell turnover (Power et al. 2025; McEachron and Helman 2021). These features may limit the ability of tumors to form persistent, functionally integrated networks, instead favoring a rapid expansion strategy that circumvents the organism's control mechanisms. By contrast, in adult cancers, the progressive deterioration of the microenvironment (Capp 2017; Capp and Thomas 2021) may create conditions that favor the emergence and stabilization of complex tumor architectures. This difference suggests that the extent to which selection for function operates in cancer is contingent on both tissue context and organismal age. This reinforces the idea that selection for function is most likely to emerge in settings where tissue dynamics slow down, allowing structurally organized configurations to persist long enough to undergo functional refinement. This shift is often accompanied by changes in extracellular matrix composition, immune system interactions, and vascular remodeling, all of which contribute to the stabilization of organized pathological structures. The degree of tissue turnover and microenvironmental plasticity may therefore serve as key determinants of whether selection for function can shape disease progression.

These insights emphasize the need for distinct therapeutic strategies. In adult cancers where selection for function plays a key role, disrupting functional tumor networks may be critical to counteract persistence. Conversely, in pediatric malignancies, where rapid expansion dominates (driven by cell‐level selection), targeting developmental pathways and cellular plasticity may represent more effective therapeutic avenues. Beyond pediatric cancers, other pathologies affecting highly dynamic tissues may similarly represent settings where selection for function is less prominent, not because microenvironmental constraints are absent, but because rapid tissue turnover and repeated damage‐repair cycles prevent the long‐term stabilization of specific pathological configurations. Such conditions, including chronic inflammatory diseases or fibrotic lesions in highly dynamic tissues, often involve repeated cycles of damage and repair. While injury may promote lesion initiation, frequent tissue remodeling and turnover can limit the long‐term stabilization of specific pathological structures, thereby reducing opportunities for selection for function to operate. This distinction aligns with broader patterns observed in medicine: acute diseases often progress rapidly without the establishment of persistent structural configurations, whereas chronic conditions, such as cancer, fibrosis, or neurodegeneration, tend to exhibit increasing complexity and resistance, shaped by functional selection. Crucially, for such a selective process to operate, many precursor structures must initially form and subsequently fail, with only a minority persisting. Importantly, this failure rate is likely to decrease with advancing age, as the progressive decline of maintenance and clearance mechanisms provides a more permissive environment for the stabilization and accumulation of resistant pathological configurations.

In atherosclerosis, plaque development is not a passive accumulation but an active, dynamic process driven by the recruitment of inflammatory cells and vascular smooth muscle cells (VSMCs) (Watson et al. 2020; Libby 2021; Tabas et al. 2015). As discussed above, while many early plaques regress, some acquire structural features, such as fibrous caps reinforced by VSMCs, that promote their persistence within the arterial wall. This phenomenon exemplifies well selection for function, where the plaque evolves to maintain its structural integrity and resist hemodynamic stresses, even at the expense of long‐term cardiovascular health. It is important to clarify that what “evolves” in this context is not the plaque itself as an independent entity, but rather the specific structural configurations that differentially persist due to their mechanical and biological advantages. These orchestrated interactions lead to the formation of structured fibrotic caps that stabilize the plaque and ensure its persistence within the arterial wall. The stabilization process involves VSMCs migrating to the intima, proliferating, and depositing extracellular matrix components, thereby reinforcing the fibrous cap. While this structural fortification reduces the immediate risk of plaque rupture, it contributes to chronic luminal narrowing and impaired blood flow, highlighting a trade‐off between plaque stability and overall arterial function and organismal health. Understanding this adaptive mechanism points to potential therapeutic strategies aimed at modulating plaque composition and stability to mitigate adverse cardiovascular outcomes.

Similarly, in neurodegenerative diseases such as Alzheimer's, amyloid‐beta plaques and tau neurofibrillary tangles are not mere byproducts, but evolve into structured entities that actively disrupt neuronal function (Rahman and Lendel 2021). These aggregates interfere with cellular clearance mechanisms, resist degradation, and persist within the brain's microenvironment, suggesting an evolved stability that complicates their elimination. Amyloid‐beta plaques arise from the extracellular accumulation of misfolded amyloid‐beta peptides, forming dense structures that impair synaptic communication and trigger inflammatory responses. In parallel, tau proteins undergo hyperphosphorylation, generating intracellular tangles that destabilize microtubules and compromise neuronal transport systems (Spires‐Jones and Hyman 2014). The resilience and structural integrity of these protein aggregates provide yet another example of selection for function. Like other pathological entities, amyloid plaques and tau tangles persist and self‐organize in ways that enhance their stability, even at the expense of neuronal function and overall cognitive health. However, these aggregates do not evolve as autonomous units but rather follow a process of preferential stabilization, whereby multiple precursor structures initially form, but only those configurations that resist clearance persist and accumulate over time (Spires‐Jones and Hyman 2014; Rahman and Lendel 2021). Their ability to evade degradation and propagate within the brain reflects a selection process favoring persistence at the lower level over immediate cellular fitness. This perspective suggests that neurodegenerative aggregates, much like tumors and atherosclerotic plaques, are shaped by selection for function at a lower level. This insight has critical therapeutic implications, as it suggests that treatments should not only aim to eliminate protein aggregates but also to disrupt the functional networks sustaining their persistence. By targeting the stability mechanisms that drive their accumulation and self‐organization, novel therapeutic strategies could be developed to prevent the reinforcement of pathological configurations, ultimately improving cognitive outcomes in affected individuals. Interestingly, early‐onset neurodegenerative diseases, such as juvenile ALS (amyotrophic lateral sclerosis) or familial Parkinson's disease, may represent exceptions, where selection for function plays a limited role due to the rapid progression of disease, which prevents the establishment of highly structured pathological aggregates.

## 3 How This Model Differs From Existing Theories

Existing evolutionary models of aging, such as antagonistic pleiotropy and mutation accumulation, focus primarily on explaining why aging evolved, attributing the rise of age‐related chronic diseases to declining forces of natural selection. While these models correctly capture key aspects of the evolution of aging, they do not explain why certain pathological entities become increasingly structured and persistent over an individual's lifetime. Other emerging frameworks, such as the developmental theory of aging, which combines both hyperfunction and non‐adaptive models of late‐life dysfunction (i.e., hyper‐ and hypofunction) (Gems 2022; Lemaître et al. 2024), further emphasize the active role of growth‐related biological programs or developmental constraints in driving late‐life dysfunction. While our approach aligns with these insights, it seeks to address a complementary question: how and why do certain pathological structures, including non‐replicative ones, become increasingly functionally organized, persistent, and resilient over time? By refining this concept, we emphasize that in addition to selection processes acting on individuals (and affecting the genetic make‐up of populations), selection for function acting at lower organizational levels can favor the persistence of specific structural configurations, which become preferentially stabilized under local environmental constraints and internal feedback mechanisms. This hypothesis offers a key distinction: rather than viewing aging‐related diseases simply as a passive consequence of long‐term weakened selection against alleles with late‐in‐life detrimental effects at the organismal level, it suggests that these conditions evolve actively through selection for function at the lower level, facilitated by the relaxation of selection at the organismal level late in life. Unlike developmental or genetic theories that emphasize continued expression or deregulation of early‐life programs, our model highlights how pathological configurations may undergo internal filtering and reinforcement, regardless of their genetic origin. This perspective integrates both the loss of selective constraints at the organismal level and the emergence of alternative selective pressures at the lower level, providing a more complete evolutionary framework. It explains why pathological structures become functionally organized, resilient to degradation, and resistant to therapeutic interventions, a dynamic largely overlooked in classical aging theories. Moreover, this model fits within a multi‐level selection (MLS) framework, where Darwinian selection continues to shape the organismal and cellular levels, while selection for function governs the organization of non‐replicative pathological structures, such as tumors and amyloid plaques. By shifting the focus from how selection declines at the organismal level to what selective forces take over at lower (including non‐replicative entities), this hypothesis opens new avenues for both evolutionary biology and medical research.

## 4 Age‐Specific Expression of Pathological Structures: A Signature of Selection for Function

Interestingly, this framework may also help explain a striking epidemiological pattern observed across many chronic diseases: the same types of structural pathologies that are extremely rare in young individuals often become frequent in older adults. Conditions such as cataracts, osteochondral lesions, valvular heart diseases, and tissue calcifications illustrate this pattern. In young individuals, these pathologies are typically associated with rare developmental mutations or congenital malformations, which often impose a strong fitness cost, impacting survival, growth, or reproductive potential (Stephen 2015; Hamilton 1966; Kirkwood 1977). Under such circumstances, natural selection acts strongly to eliminate these traits from the population. By contrast, in older individuals, after the decline of natural selection (Hamilton 1966; Kirkwood 1977), these same types of structures frequently emerge, not through genetic mutations, but through progressive tissue remodeling and the local action of selection for function. Behaviors and exposures, as to cigarette smoke, can further corrupt tissue microenvironments and evolved constraints, thereby facilitating the emergence and persistence of pathological configurations. Such environmental insults not only increase mutation rates but also promote chronic inflammation, tissue remodeling, and microenvironmental instability, all of which can enhance the selection for function at the lower level and ultimately elevate the risk of diseases like cancer and cardiovascular conditions. In these contexts, the ability of pathological configurations to persist, stabilize, and resist clearance mechanisms becomes a key driver of their development. This pattern is consistent with the hypothesis that many chronic diseases of aging do not simply result from passive tissue degradation but rather from an active evolutionary dynamic within internal systems, involving non‐replicative units, where selection for function promotes the persistence of specific structural pathological organizations that would be strongly counter‐selected during early life.

From a therapeutic perspective, understanding the role of selection for function opens new possibilities beyond conventional strategies aimed at eliminating disease symptoms or proliferative cells. Instead of solely targeting individual molecular components or cell types, interventions could be designed to disrupt the functional integrity and internal organization of persistent pathological structures. For instance, in atherosclerosis, treatments might not only aim to reduce lipid accumulation or inflammation but also interfere with the stabilization mechanisms of fibrotic caps, such as targeting extracellular matrix components or signaling pathways involved in cap reinforcement. In Alzheimer's disease, therapies could shift from focusing solely on aggregate clearance to preventing the consolidation of amyloid‐beta into resilient structures, potentially by modulating protein conformation, phase separation dynamics, or microglial selection thresholds. As outlined before, in cancer, beyond eliminating proliferative cells, therapeutic strategies could be designed to destabilize oncogenic group phenotypic compositions by targeting the collective traits that promote tumor persistence, such as cooperative signaling, metabolic interdependence, or immune evasion at the group level. These approaches emphasize functional destabilization as a therapeutic goal, aiming to prevent pathological systems from reaching stable, self‐sustaining configurations. Importantly, such strategies may be especially relevant in aged tissues where the decline in clearance mechanisms facilitates the persistence of organized disease structures.

While our hypothesis builds upon the theoretical framework of selection for function introduced by Wong and colleagues (Wong et al. 2023), it departs significantly from their generalized claims by focusing on concrete biological systems within aging organisms. We do not propose to replace Darwinian principles acting on self‐replicating entities (organisms, cells), which already offer a comprehensive theoretical framework for the evolution of the aging process (see introduction), but to complement them, particularly when declining organism‐level selection with age interacts with internal dynamics (including acting on non‐self‐replicating units) allowing the emergence of persistent and structured pathological entities. Our approach is rooted in biological realism and public health concerns: we apply the concept to non‐replicative configurations such as amyloid plaques, fibrotic lesions, or benign tumors, whose progression and stabilization are well‐documented but insufficiently explained by classical evolutionary theories. In this context, selection for function is used as a lens to understand how certain configurations resist clearance and progressively acquire functional organization, not through heritable genetic change, but through local persistence and internal filtering.

Importantly, we distinguish our contribution from the more expansive claims of Wong and colleagues by focusing on the within‐organism scale, and by grounding our hypothesis in age‐related physiological decline, microenvironmental dynamics, and clinical examples. We thus propose a biologically bounded application of selection for function, which integrates known features of age‐related disease progression and therapeutic resistance into an evolutionary perspective. This, we believe, represents a novel and useful extension of the original framework.

While our hypothesis draws conceptual inspiration from classical within‐generation selection processes, we emphasize that the persistence of pathological structures in aging organisms involves more than passive filtering. These entities, such as plaques, aggregates, or fibrotic lesions, not only resist elimination but often acquire increased structural complexity and internal organization over time. This progressive consolidation is shaped by selective pressures within the aging microenvironment, including immune interactions, mechanical constraints, and resource gradients. Although these dynamics do not involve self‐replication and heritable genetic change, they reflect a form of intra‐organismal functional selection, where specific self‐emerging configurations are differentially selected due to their capacity to persist. This process, which we frame as selection for function, does not constitute Darwinian evolution per se but represents an internal, self‐reinforcing dynamic that leads to increasingly resilient pathological systems. By distinguishing this process from both classical damage accumulation and passive selection, we aim to highlight its potential clinical significance, particularly the need to identify and disrupt the features that confer persistence and organization to disease structures in aging tissues.

This dynamic can be usefully conceptualized through the lens of birth and death rates, allowing for a relative “fitness” interpretation of persistent structures. For instance, in the case of atherosclerotic plaques, diverse micro‐aggregates may form regularly, but in youth, most are efficiently eliminated, resulting in a net death rate exceeding the formation rate. With age, clearance efficiency declines, and certain configurations begin to persist longer than others, leading to a shift where their effective “birth rate” exceeds their “death rate” (note that “birth” refers to “spontaneous emergence” not replication). However, the configurations that persist are those that may possess properties, such as compactness, biochemical shielding, or integration into fibrotic tissue, that confer relative stability. Over time, internal rearrangements or feedback interactions can further enhance their persistence, resulting in a form of within‐organism selection and evolution where specific pathological entities gradually dominate due to higher relative “fitness”.

## 5 Conclusion

The concept of selection for function provides a novel evolutionary framework for understanding why chronic diseases of aging exhibit increasing structural organization and persistence over a lifetime. While classical aging theories emphasize the declining influence of natural selection at the organismal level, our hypothesis suggests that aging‐related diseases are not simply passive consequences of this decline but also the expected action of selection for function at lower (including non‐replicative) levels within internal biological systems. This perspective opens new avenues for therapeutic intervention. Currently, the Geroscience approach argues that, rather than merely targeting individual diseases, biogerontological research should focus on interventions, such as pharmacological strategies (e.g., (Blagosklonny 2006)), that act on fundamental molecular mechanisms and improve both healthspan and lifespan by simultaneously delaying the onset of multiple chronic conditions (Kennedy et al. 2014; Sierra 2016). While we fully concur with this mechanistic view, our evolutionary perspective highlights the need for complementary therapeutic strategies aimed at disrupting the system‐level organization of age‐associated pathological entities. By modifying the selective landscape and targeting the persistence mechanisms that sustain chronic diseases, future therapies may offer more effective solutions than current approaches focused solely on cellular elimination. Given that late‐onset diseases such as cancer can further reinforce the aging process, for example, through energy allocation trade‐offs between tumor control and other somatic maintenance mechanisms (Jacqueline et al. 2017), disrupting the functional organization of pathological entities might enable organisms to reallocate resources toward repair processes, ultimately creating a virtuous circle that slows down aging itself. This framework invites a broader reconsideration of what constitutes evolutionary adaptation in aging systems, not merely at the level of the genome or cell, but in the functional architecture of persistence itself.

## Author Contributions

All authors contributed to the development of the core idea presented in this manuscript. Antoine M. Dujon, Klara Asselin, Aurora M. Nedelcu, and Frédéric Thomas co‐wrote the first draft. Jean‐François Lemaître, Jean‐Pascal Capp, Pascal Pujol, Beata Ujvari, and James DeGregori contributed to the subsequent versions of the manuscript by providing critical insights, suggestions, and substantive revisions. All authors reviewed and approved the final version of the manuscript.

## Conflicts of Interest

The authors declare no conflicts of interest.

## Data Availability

Data sharing is not applicable to this article as no new data were created or analyzed in this study.
